# ArcGIS-based protocol to calculate the area fraction of landslide for multiple catchments

**DOI:** 10.1016/j.mex.2023.102064

**Published:** 2023-02-10

**Authors:** Naoyuki Yoshihara

**Affiliations:** Geological Survey of Japan, National Institute of Advanced Industrial Science and Technology, 1-1-1 Higashi, Tsukuba, Ibaraki 305-8567, Japan

**Keywords:** ArcGIS, Geography, Geomorphology, Hydrology, ModelBuilder, Watershed, CA, Catchment area, LA, Landslide area, LCR, The area percentage of landslides in catchment, Calculation of the area fraction of landslide for multiple catchments by using ArcGIS

## Abstract

The area fraction of specific kinds of information in a catchment provides parameters to be utilized in catchment-scale analyses. For example, the area fraction of soil movement caused by landslides is an indicator for the estimation of the magnitude of landslides. However, catchment-scale analyses often require applying the same processing to higher numbers of study catchments, making it a time-consuming process. Here an ArcGIS-based method has been presented to reduce cumbersome procedures for the calculation of the area fraction of several target surface data. The method applies automated and iterative processing to multiple catchments, whose location and scale are defined by users. This method may prove to be useful for calculating the area fraction of parameters other than landslide area (e.g., specific land use or lithology) within a framework of catchment-scale analysis.

• An Arcgis-based method to calculate the area fraction of landslide area in catchments.
• Manual work is reduced by automated and iterative processing based on ModelBuilder.
• It can be used to get the area fraction of several surface information in catchments.

## Graphical abstract

**Figure:**
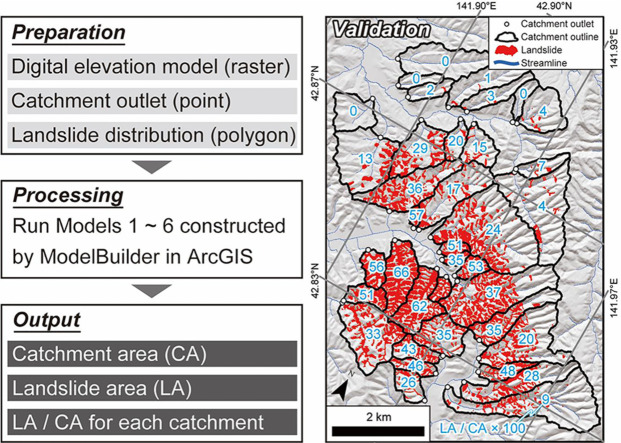

Specifications table

**Table:** 

Subject area:	Earth and Planetary Sciences
More specific subject area:	Geography
Name of your method:	Calculation of the area fraction of landslide for multiple catchments by using ArcGIS
Name and reference of original method:	N. Yoshihara, S. Matsumoto, R. Umezawa, I. Machida, Catchment-scale impacts of shallow landslides on stream water chemistry. Sci. Total Environ. 825 (2022) 153970. https://doi.org/10.1016/j.scitotenv.2022.153970.
Resource availability:	ArcGIS Pro ver. 2.7 (or later) with Advanced License

## Method details

This method was designed to calculate the percentage of landslide area (LA)/catchment area (CA) for 37 forested catchments in Hokkaido, Japan, where shallow landslides (i.e., destabilization of soil-mantled hillslope) occurred due to the Hokkaido Eastern Iburi earthquake in 2018 (6.6 Mw). However, this method can also be used to obtain the area fraction of surface information other than LAs (e.g., specific kinds of land use or lithology) within catchments.

The method requires an operator to use the ArcGIS software with an advanced license and prepare surface raster data of digital elevation and a shapefile of landslide polygons. The procedure consists of six models based on the ModelBuilder utility in ArcGIS. The resulting output visualizes catchment outlines and landslide distribution within the study catchments and calculates the CA, LA, and area percentage of landslide scar (LCR) described as LA/CA × 100.

### Model 1

**Fig. 1:**
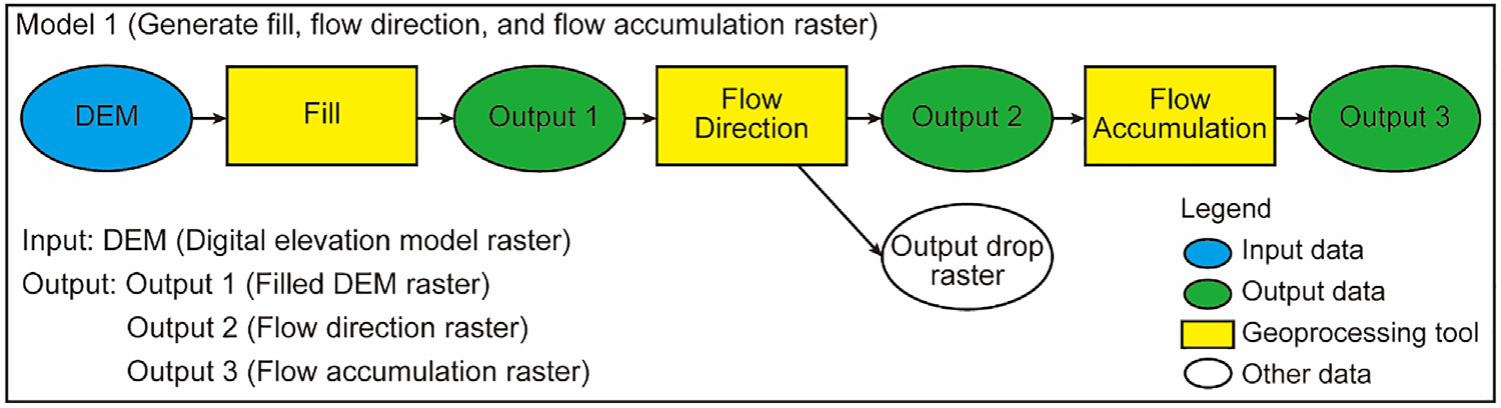
Workflow of Model 1.

The workflow of Model 1 based on the ModelBuilder utility is described in Fig. 1. Model 1 removes small sinks or imperfections in the digital elevation model (DEM) and visualizes the streamline. This model generates a flow direction and a flow accumulation raster. These two raster data are used to generate a watershed raster in Model 2 and to determine the catchment outlet in the manual procedure described in the following section.

### Manual procedure—determination of catchment outlets

After running Model 1, certain manual operations are required. Primarily, add the flow accumulation raster to the map window in ArcGIS. Further, edit the symbology of the flow accumulation raster to visualize the streamline. For example, set the symbology of the flow accumulation raster as follows: the primary symbology is “Classify”; the method is “Manual Interval”; the number of the class is “2”; a symbol for a value less than 1000 has no color (transparent) and another color depends on the operator's preference. The above-mentioned settings will lead to visualization of the streamline. Then, create a shapefile containing points that represent outlets of the study catchments. Here the catchment outlets must be placed on the streamline shown in the flow accumulation raster for a successful creation of the watershed raster in Model 2.

### Model 2

**Fig. 2:**
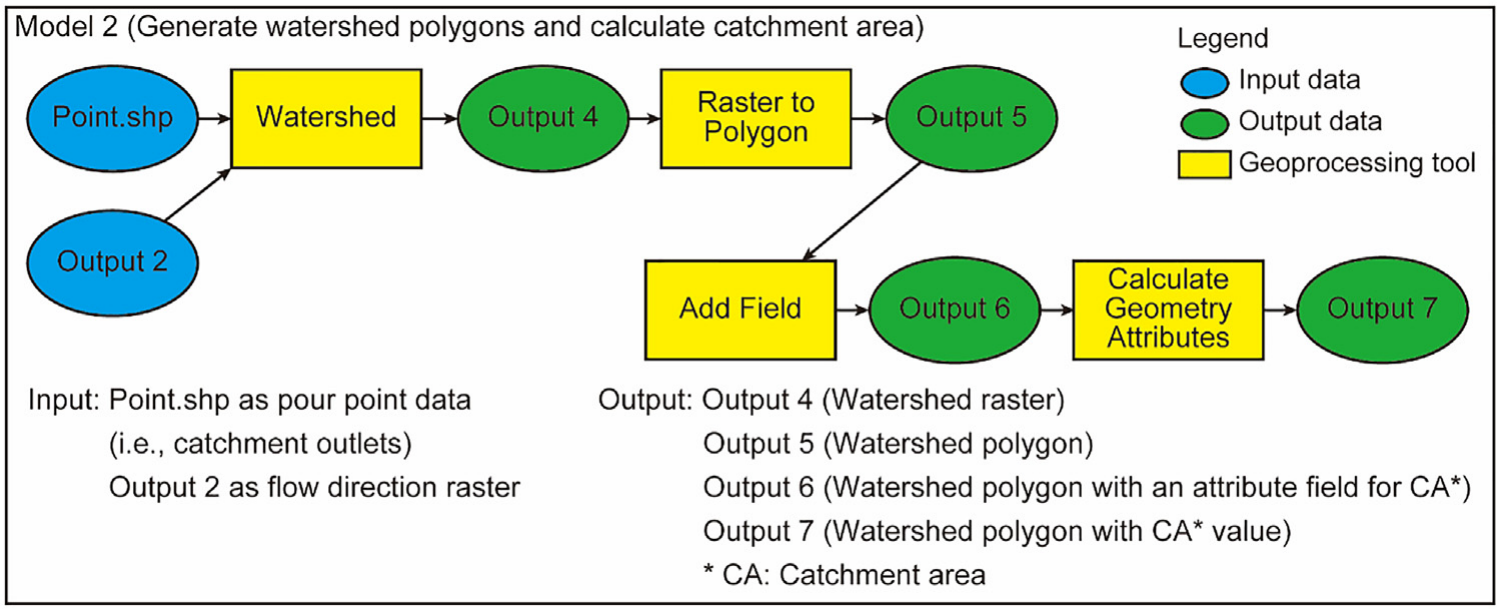
Workflow of Model 2.

Model 2 generates a raster and a shapefile exhibiting outlines of target catchments (Fig. 2). After generating the raster and shapefile, the CA is calculated on each catchment polygon. This model first generates a watershed raster from the points of the catchment outlet and the flow direction raster by using the “Watershed” tool. Further, the watershed raster is converted to a shapefile that stores watershed polygons by the “Raster to Polygon” tool. Ultimately, Model 2 adds a new attribute field for the CA to a table of the watershed polygon using the “Add Field” tool and calculates CA values using the “Calculate Geometry Attributes” tool.

### Model 3

**Fig. 3:**
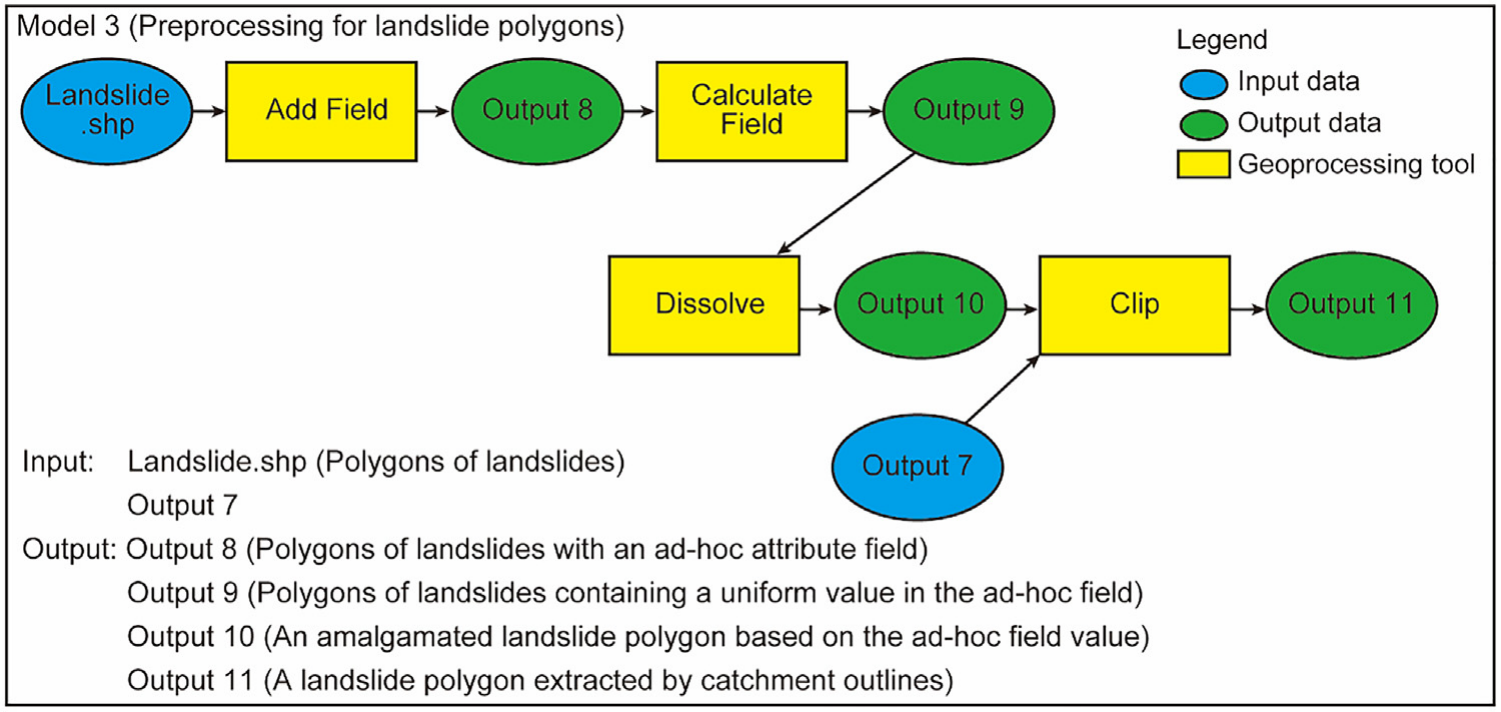
Workflow of Model 3.

Model 3 preprocesses landslide polygons inside each catchment outline for automated and iterative extraction (Fig. 3). Duplication of landslide polygons is removed by executing Model 3. In addition, all of the landslide polygons are merged into a single polygon. The merged landslide polygon is saved as a single shapefile. Model 3 uses the “Add Field” and “Calculate Field” tools to add an ad-hoc attribute field with a uniform value to the landslide polygons. For example, the integer “9999” may be applicable to be written in the ad-hoc field for all polygons by using the “Calculate Field” tool. The landslide polygons are then amalgamated based on the ad-hoc field using the “Dissolve” tool. The dissolved landslide polygon overlaying any of the catchment polygons is then extracted by the “Clip” tool.

### Model 4

**Fig. 4:**
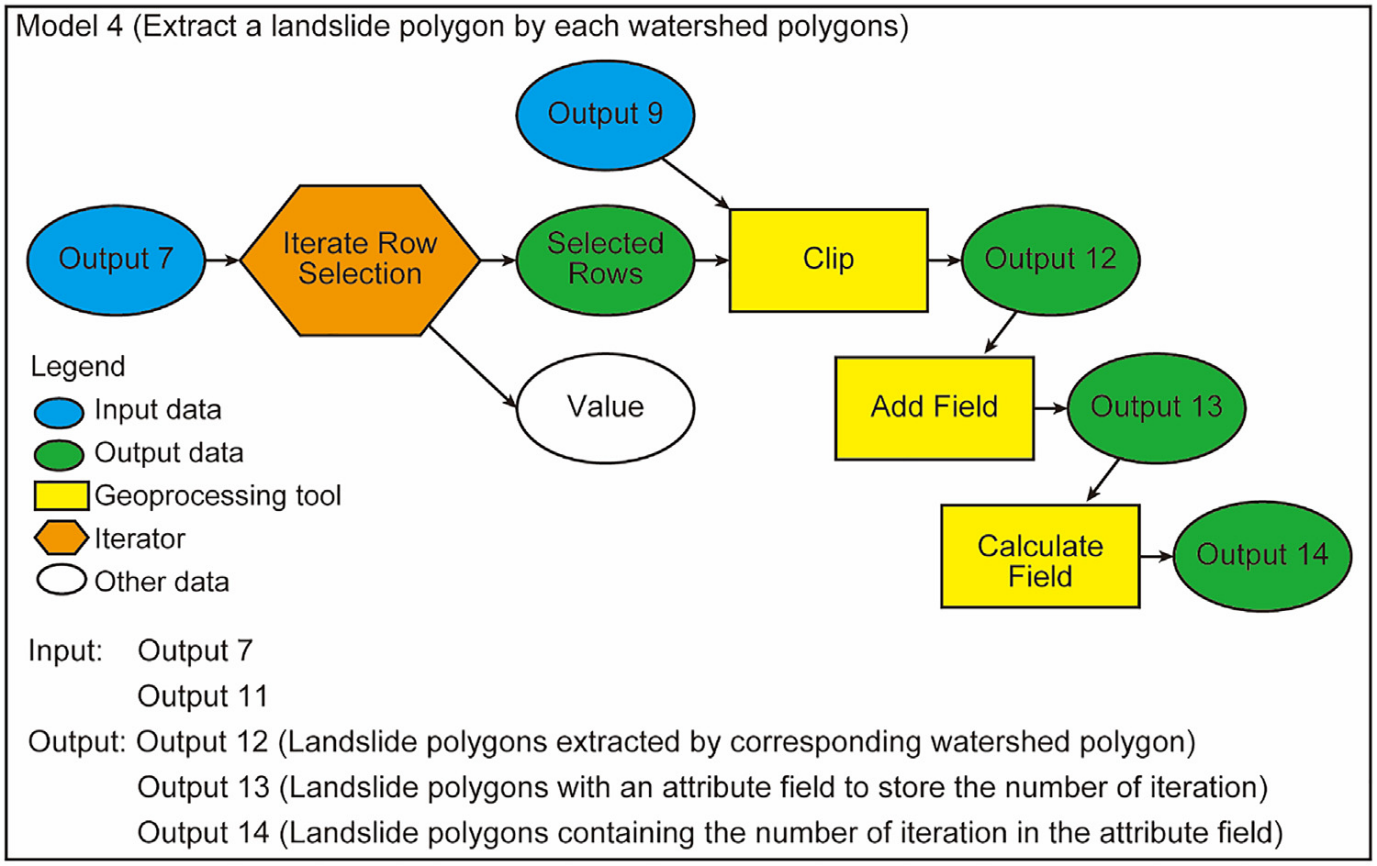
Workflow of Model 4.

Model 4 carries out iterative extraction of the landslide polygon overlaying each catchment (Fig. 4). This processing is done by combining the iterator of “Iterate Row Selection” and the geoprocessing tool of “Clip”. Iterator functionalities can be used in ModelBuilder. Here the clipped shapefiles of landslide polygons must be saved in the same file geodatabase and named differently for the following processes. Therefore, it is recommended to set the name of output features such as “Landslide_%Value%”, where the text “%Value%” stores the number of iterations automatically generated by the “Iterate Row Selection” functionality. In this paper, “%Value%” stores the “OBJECT ID” of the selected watershed polygon. The OBJECT ID, which is an integer starting from “1”, is automatically assigned to the attribute field of the watershed polygons when the “Raster to Polygon” tool was run in Model 2. The model thus iteratively selects a catchment polygon and generates shapefiles containing a landslide polygon within the selected catchment polygon. These shapefiles are named with a sequential number (e.g., “Landslide_1.shp”, “Landslide_2.shp”, and “Landlide_3.shp”).

Executing the “Add Field” and “Calculate Field” tools on the landslide polygons that were extracted by the “Clip” tool, an attribute field containing a variable “%Value%” is attached to each shapefile of a clipped landslide polygon (Fig. 4). This field value is used in Model 6 to write LA values on attribute tables of the watershed polygons.

### Model 5

**Fig. 5:**
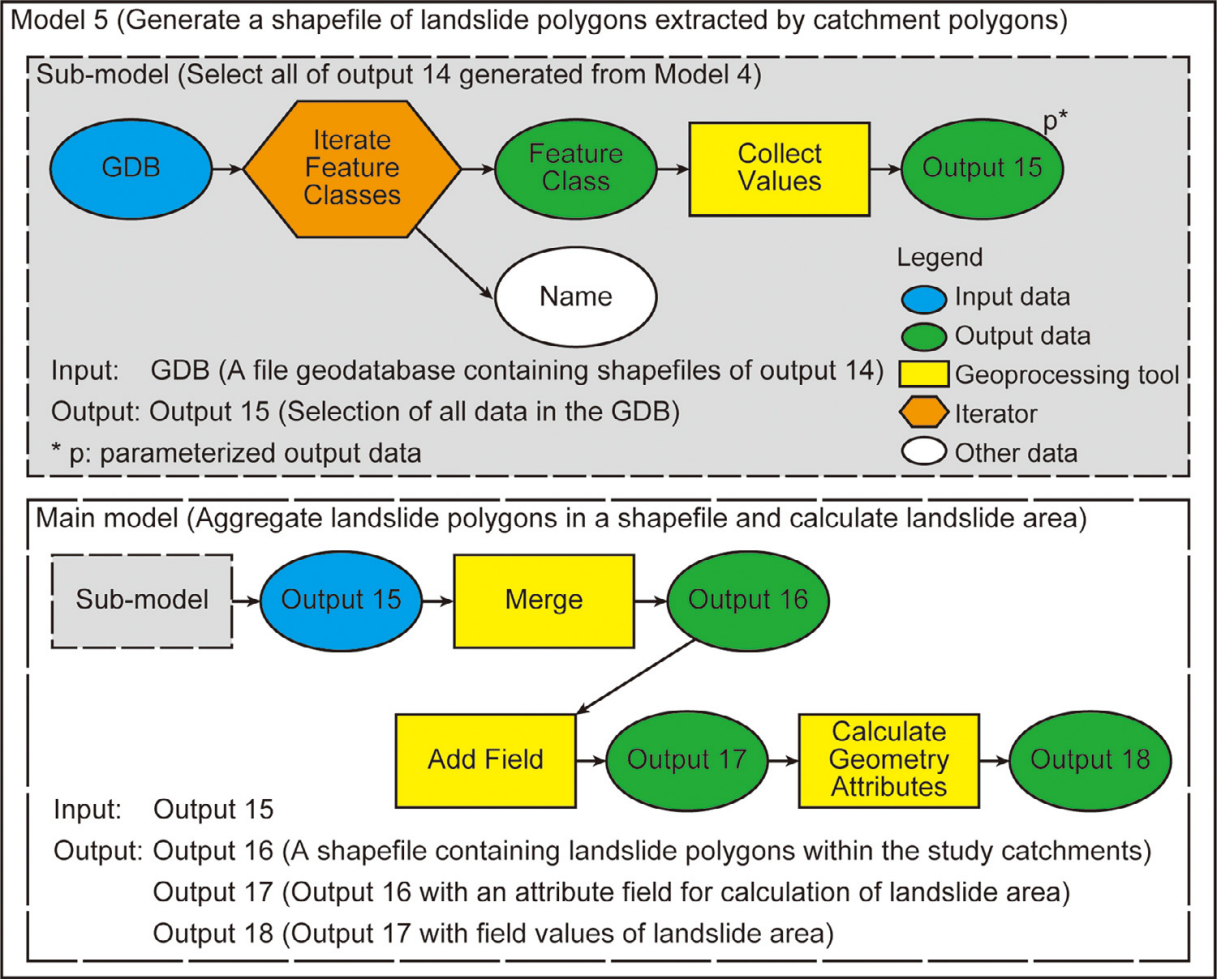
Workflow of Model 5.

Model 5 contains a sub-model and uses the output data generated from it (Fig. 5). In the sub-model, output data of Model 4 (i.e., a suite of landslide polygons overlaying individual catchment polygons) are selected. A combination of an iterator “Iterate Feature Classes” and a utility “Collect Values”, both of which are used in ModelBuilder, enables a sequential selection of the landslide polygons. Output data generated by the “Collect Values” utility is assigned as a parameter to be used as input data in Model 5.

After running the sub-model and getting the output data as a parameter, the main model in Model 5 applies the “Merge” tool to generate a shapefile containing the collected feature classes. Next, the main model runs the “Add Field” tool to add a new field “LA” to an attribute table of the landslide polygons generated by the “Merge” tool. Finally, the LA for each catchment is calculated by the “Calculate Geometry Attributes” tool.

### Model 6

**Fig. 6:**
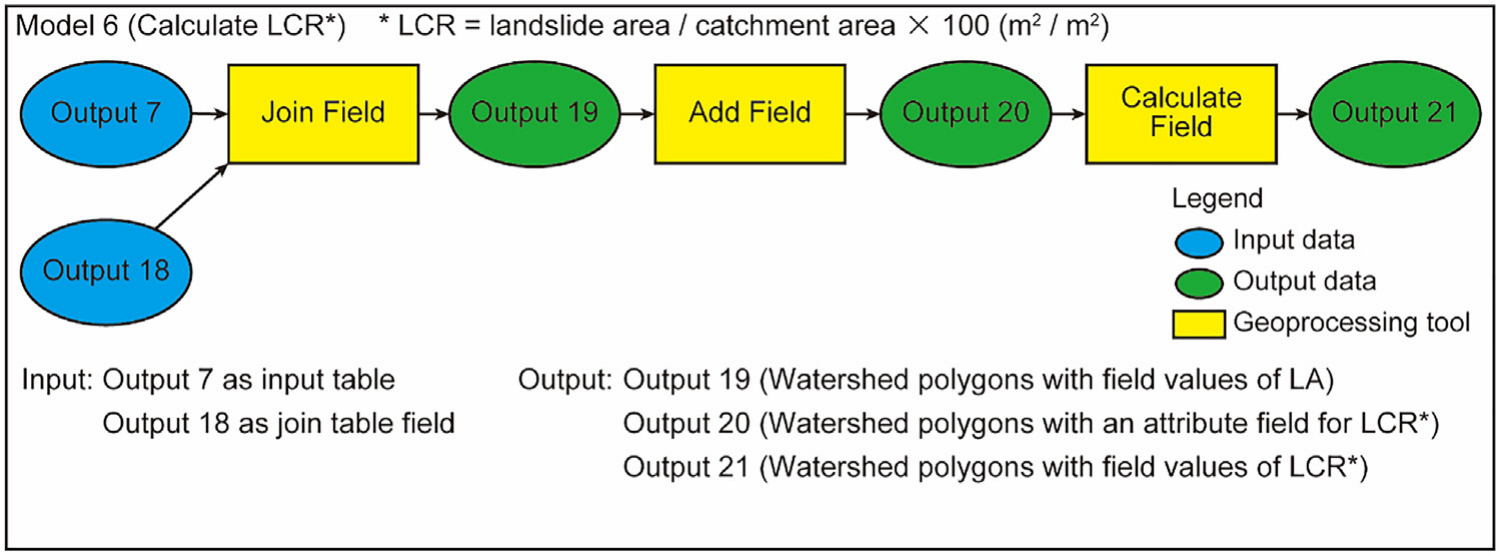
Workflow of Model 6.

Model 6 obtains target parameters: CA, LA, and LCR in this paper (Fig. 6). The calculation is performed in an attribute table of the watershed polygons. The “Join Field” tool transfers LA values from the attribute table of the merged landslide polygons to that of the watershed polygons. Next, a new field “LCR” is added to the attribute table of the watershed polygon by the “Add Field” tool. Finally, LCR (LCR = LA/CA * 100) is calculated by the “Calculate Field” tool. Here CA, LA, and LCR are obtained for the study catchments.

### Method validation

**Fig. 7:**
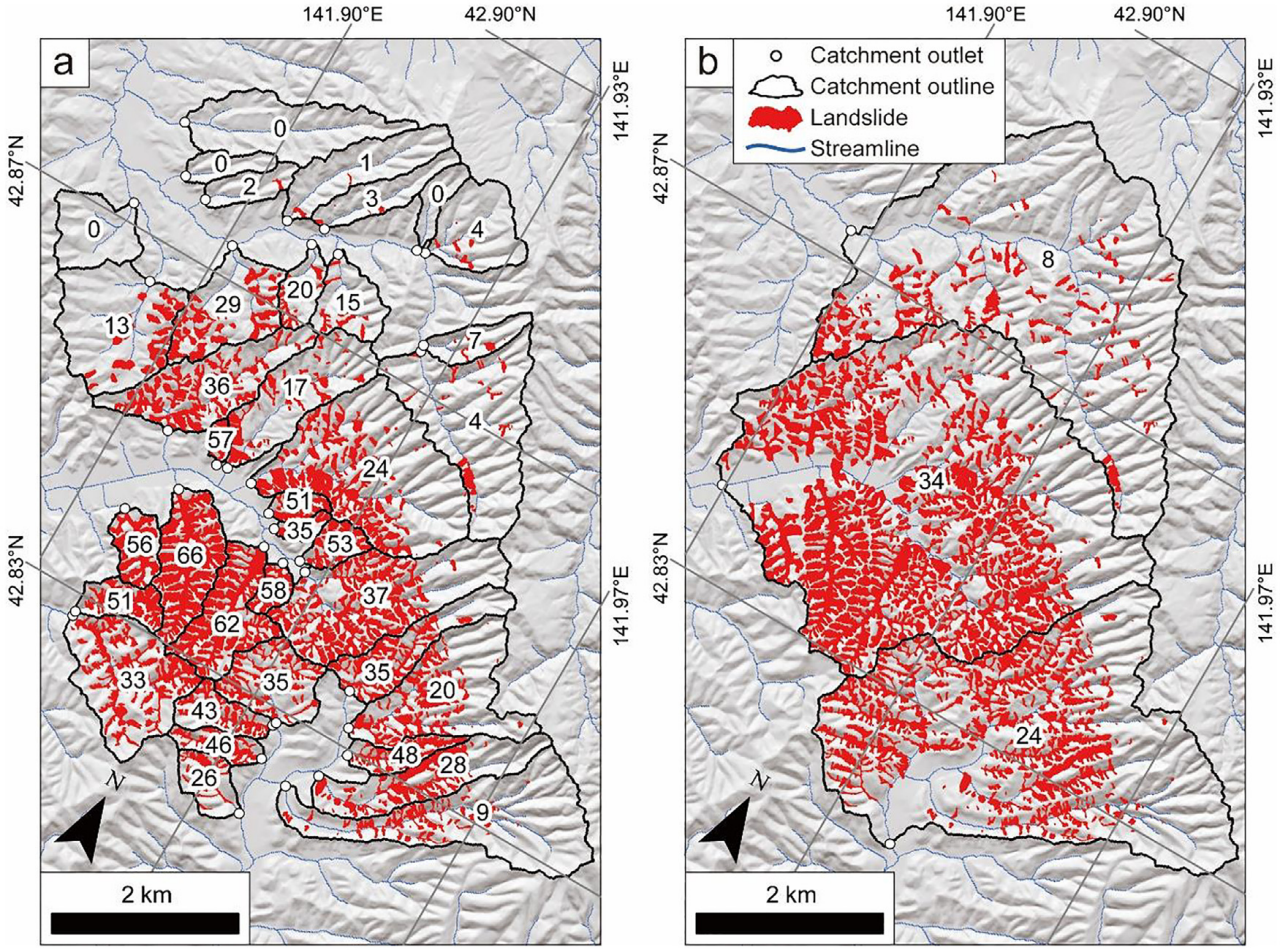
Validation results of the method used in forested catchments in Hokkaido, Japan, where shallow landslides were caused by the Hokkaido Eastern Iburi earthquake in 2018. (a) Application on 37 catchments by Yoshihara et al. [1]. (b) Application on three catchments. Open circles and black lines represent outlets and outlines of catchments, respectively. Distribution of landslides is shown in red. LCR (landslide area/catchment area × 100) is described in each catchment. The streamline is visualized by editing the symbology of the flow accumulation raster as follows: the primary symbology is “Classify”; the method is “Manual Interval”; the number of the class is “2”; the symbol for a value less than 1000 has no color (transparent); another color is blue.

The presented method was applied to forested catchments in southern Hokkaido, Japan [1]. In this region, more than 6000 shallow landslides were caused by the Hokkaido Eastern Iburi earthquake in 2018 [2,3]. The distribution of the shallow landslides was manually defined by referring to DEMs and aerial photographs before and after the earthquake. Fig. 7a shows a result of the application of our suggested method [1]. In this case, 37 catchments were analyzed and the CA, LA, and LCR were in the range of 0.08–2.56 km^2^, 0–0.70 km^2^, and 0–66%, respectively. Editing outlet points were performed to generate a different result (Fig. 7b). In this case, three outlets of the catchment were set and the CA, LA, and LCR were in the range of 8.52–11.47 km^2^, 0.71–3.91 km^2^, and 8–34%, respectively.

## Related research article

**For a published article:** N. Yoshihara, S. Matsumoto, R. Umezawa, I. Machida, Catchment-scale impacts of shallow landslides on stream water chemistry, Sci. Total Environ. 825 (2022) 153970. https://doi.org/10.1016/j.scitotenv.2022.153970.

## Funding

This research did not receive any specific grants from funding agencies in the public, commercial, or not-for-profit sectors.

## CRediT authorship contribution statement

**Naoyuki Yoshihara:** Conceptualization, Methodology, Formal analysis, Validation, Visualization, Writing – original draft, Writing – review & editing.

## Declaration of Competing Interest

The authors declare that they have no known competing financial interests or personal relationships that could have appeared to influence the work reported in this paper.

## Data Availability

Data will be made available on request.
